# Two Low Coverage Bird Genomes and a Comparison of Reference-Guided versus *De Novo* Genome Assemblies

**DOI:** 10.1371/journal.pone.0106649

**Published:** 2014-09-05

**Authors:** Daren C. Card, Drew R. Schield, Jacobo Reyes-Velasco, Matthew K. Fujita, Audra L. Andrew, Sara J. Oyler-McCance, Jennifer A. Fike, Diana F. Tomback, Robert P. Ruggiero, Todd A. Castoe

**Affiliations:** 1 Department of Biology, The University of Texas at Arlington, Arlington, Texas, United States of America; 2 United States Geological Survey – Fort Collins Science Center, Fort Collins, Colorado, United States of America; 3 Department of Integrative Biology, University of Colorado Denver, Denver, Colorado, United States of America; 4 Department of Biochemistry and Molecular Genetics, University of Colorado School of Medicine, Aurora, Colorado, United States of America; Fordham University, United States of America

## Abstract

As a greater number and diversity of high-quality vertebrate reference genomes become available, it is increasingly feasible to use these references to guide new draft assemblies for related species. Reference-guided assembly approaches may substantially increase the contiguity and completeness of a new genome using only low levels of genome coverage that might otherwise be insufficient for *de novo* genome assembly. We used low-coverage (∼3.5–5.5x) Illumina paired-end sequencing to assemble draft genomes of two bird species (the Gunnison Sage-Grouse, *Centrocercus minimus*, and the Clark's Nutcracker, *Nucifraga columbiana*). We used these data to estimate *de novo* genome assemblies and reference-guided assemblies, and compared the information content and completeness of these assemblies by comparing CEGMA gene set representation, repeat element content, simple sequence repeat content, and GC isochore structure among assemblies. Our results demonstrate that even lower-coverage genome sequencing projects are capable of producing informative and useful genomic resources, particularly through the use of reference-guided assemblies.

## Introduction

High quality sequencing, assembly, and annotation of vertebrate genomes have become feasible for non-traditional model species, as costs of sequencing decrease and analysis methods improve. The default method for generating initial genome assemblies for a species includes the use of *de novo* assembly algorithms that rely on sufficient overlap between sequencing reads to build larger contiguous sequences. This approach is fundamentally different from a reference-guided approach that utilizes existing contiguous sequences and sequence similarity between the target and reference species' genomes to assemble a genome. The availability of high quality reference genomes for a greater diversity of vertebrate species may enable inexpensive yet informative genomic resources to be generated for new species by leveraging information from existing high-quality genomes of related species. If there is a relatively high degree of synteny among related species, a reference-guided genome assembly approach may be capable of delivering more complete and biologically useful genome resources with far less data and computational effort than required for full *de novo* genome assembly. Thus, we may potentially achieve greater representation and understanding of genomic diversity across the tree of life through the use of high-quality genomes, complemented by the addition of lower-coverage genomes.

Among amniote vertebrates, birds possess among the smallest genomes and the lowest levels of repetitive elements [1]–[3]. These two characteristics make their genomes relatively inexpensive to sequence and also make mapping and assembling genomic sequencing reads computationally more tractable. Bird genomes are also highly conserved at the chromosomal level, such that there is a high degree of synteny across chromosomes of divergent bird species [4]–[6]. This karyotypic conservation facilitates ready transfer of information from one bird genome to another [7]–[9] and justifies their use as a system to test a reference-guided genome assembly approach in this study. Birds are important model systems for a broad diversity of research, and having genomic information to facilitate these diverse research programs for all bird species would be ideal, which motivates the development of efficient and inexpensive means of assembling genomes and genomic resources. This raises the questions: 1) Can low coverage sequencing of new bird genomes be used to economically produce biologically valuable genome resources by leveraging existing complete genomes, and 2) How does the content of different types of biological features (e.g., genes, transposable elements, and GC-isochores) compare among low coverage *de novo*, low coverage reference-guided, and existing high-coverage high quality genomes?

In this study we use existing high-quality bird genomes from the Chicken (*Gallus gallus*; [1]) and the Zebra Finch (*Taeniopygia guttata*; [3]) to guide the assembly of two distantly related bird species, the Gunnison Sage-Grouse (*Centrocercus minimus*; Order Galliformes, Family Phasianidae, “Sage-Grouse” hereafter) and the Clark's Nutcracker (*Nucifraga columbiana*; Order Passeriformes, Family Corvidae, “Clark's Nutcracker” hereafter). For the purposes of this study, we define a high-quality reference genome as a genome with N50 contig lengths of >10kb that have been ordered and combined into supercontigs (or scaffolds). Ideally a high-quality genome would also have >200 Mb scaffolds, which are mapped to physical chromosomes (as is the case with the two bird reference genomes used here). The Clark's Nutcracker is an important seed disperser for two widely distributed Western North American conifers, whitebark pine (*Pinus albicaulis*) and limber pine (*P. flexilis*), which are declining due to the outbreaks of the mountain pine beetle (*Dendroctonus ponderosae*) and the invasive disease white pine blister rust (*Cronartium ribicola*; [10]–[12]. Because the Clark's Nutcracker-mediated seed dispersal is key to maintaining viable populations of these imperiled pines [13], [14], knowledge of population structure and dynamics of the Clark's Nutcrackers may provide important information relevant to management of these trees. The Gunnison Sage-Grouse is a geographically restricted species of grouse found south of the Colorado River in Colorado and Utah. The entire species consists of seven small populations ranging in size from 40 birds in the smallest population to roughly 2,500 in the largest [15], [16]. Most populations are isolated from one another and have low levels of genetic diversity [17]. This species has been proposed for listing as threatened or endangered under the U.S. Endangered Species Act. The Sage-Grouse is in the order Galliformes along with the Chicken (*Gallus gallus*), for which a high quality genome is available [1]. Similarly, the Clark's Nutcracker belongs in the order Passeriformes with the Zebra Finch (*Taeniopygia guttata*), for which there is also a high-quality genome [3]. These available high-quality genomes from species related to our two species of interest present an opportunity to evaluate the utility and feasibility of reference-guided (versus *de novo*) assembly strategies.

Reference-guided genome assembly approaches have been used previously (e.g., [18]–[21]) and various pipelines currently exist for reference-guided assembly (e.g., MOSAIK – http://code.google.com/p/mosaik-aligner/; DNASTAR – http://www.dnastar.com/default.aspx). Indeed, many bacterial genomes have been generated with this approach (e.g., [18], [19]). The sequencing coverage in previous studies was, however, moderately high (>10x), and the reads were mapped to a guide genome of a very closely related species (e.g., a different strain of a species or a sister species in [21] and [20]). Here we evaluate the feasibility of using relatively low genomic coverage (∼3.5–∼5.5x) to assemble draft bird genomes using reference genomes from relatively distantly related species (>40 million years divergence between the species studied and the species' genomes used to guide the assembly; [22]–[25]). We hypothesized that with such low sequencing coverage, a traditional *de novo* assembly approach would yield a less contiguous genome with fragmentary biological features, but that a reference-guided approach might provide substantial gains in contiguity and the presence of intact biological features. Indeed, we find that the reference-guided approach substantially improves assembly and yields more informative genome assemblies as measured by most assessment metrics, indicating that this type of approach provides an economical alternative method for obtaining a preliminary estimate of genomic diversity and structure across a very large number of vertebrates.

## Materials and Methods

### Ethics Statement

Sage-Grouse blood was obtained from a single individual bird from Gunnison County, Colorado, USA, where no permit was required for trapping at the time of sampling. The trapping and sampling approach was approved and carried out by the Colorado Division of Wildlife. The Clark's Nutcracker muscle was sampled from an individual bird trapped near Logan, Utah, USA, which was kept as part of a long-term study at Northern Arizona University (IUCUC protocol 00-006) before its death from natural causes; the carcass was donated for genetic work by Alan Kamil (University of Nebraska) and Russell Balda (Northern Arizona University).

### Preparation and sequencing of shotgun sequencing libraries

The methods used to prepare and sequence shotgun libraries of the Sage-Grouse and the Clark's Nutcracker were described previously [26]. Briefly, DNA was extracted from blood (Sage-Grouse) and muscle (the Clark's Nutcracker) samples using standard phenol-chloroform-isoamyl alcohol separation and the Wizard Genomic DNA Purification Kit (Promega) respectively. Illumina paired-end libraries were prepared by fragmenting genomic DNA using nebulization, ligation of “Y”-adapters, and size selection of libraries from agarose electrophoretic gels. The libraries, including adapters, had a mean size of 325 bp and were sequenced on the Illumina GAIIx platform with 120 bp paired-end reads. Raw sequence data were deposited in the NCBI Short Read Archive (SRA Accessions SRX468855 for the Sage-Grouse and SRX468897 for the Clark's Nutcracker).

### De novo draft genome assembly

Raw read data were first demultiplexed and quality-trimmed to remove low quality reads and base calls in CLC Genomics Workbench using a modified Mott trimming algorithm and a parameter value limit of 0.05; ambiguous nucleotides were trimmed using a maximum number of ambiguities of two. *De novo* assembly was conducted in CLC Genomics Workbench using automatic word size and bubble size, and a minimum contig length of 200 bp. Paired read distances were automatically detected and contigs were scaffolded where possible. Following assembly, the reads were mapped back to the contigs using a mismatch cost  = 2, insertion cost  = 3, deletion cost  = 3, length fraction  = 0.5, and similarity fraction  = 0.8; contigs were updated and gaps were filled.

### Reference-guided draft genome assembly

We used the Chicken (*Gallus gallus* v. Galgal4; [1]) and the Zebra Finch (*Taeniopygia guttata* v. taeGut3.2.4; [3]) genomes to guide assembly of the Sage-Grouse and the Clark's Nutcracker, respectively. Quality trimmed reads from the two species in this study were mapped against their respective guide genome using CLC Genomics Workbench, with a mismatch cost  = 2, insertion cost  = 3, deletion cost  = 3, length fraction  = 0.5, and similarity fraction  = 0.8, with paired distances automatically detected. A consensus sequence for each new species was exported using different thresholds of minimum coverage for reads mapping to the consensus (1x, 2x, and 5x). For example, a 1x reference-guided assembly denotes the consensus sequence at all positions where at least one read mapped. At positions where the threshold of minimum coverage was not met, an N ambiguity was inserted. At positions where disagreements in base calls were observed between reads (with disagreements representing at least 10% of the total reads at that position, and at least two reads supporting an alternative allele), an appropriate ambiguous nucleotide symbol was inserted.

### Calculation of basic genome statistics and breaking of poly-N stretches

The reference-guided assemblies resulted in a mosaic of non-ambiguous regions interspersed with stretches of N ambiguities. Shorter stretches of N ambiguities are typical even in high quality scaffolded genome assemblies, but longer stretches (>500 bp) typically are not. Therefore, for the reference-guided assemblies we used a Perl script to break the consensus contigs at N ambiguity stretches of greater than 500 consecutive Ns. For the modified reference-guided assemblies and the *de novo* assembly, we assessed contiguity by calculating the frequency distribution of contig lengths and calculated standard statistics, such as the N50 contig length.

### Analysis of CEGMA genes and repeat element content

To assess the completeness of each assembly with regard to gene content we used the CEGMA pipeline [27], which searches assemblies for a set of core eukaryotic genes (CEGs) that are highly conserved and present in nearly all eukaryotes. The proportion of complete and partial CEGs (out of 248 possible) is taken as a measure of the completeness of the gene content of an assembly. The CEGMA pipeline was run on the *de novo* assembly, the three reference-guided assemblies, and the guide reference genomes.

Repeat elements often increase the difficulty of vertebrate genome assembly, and therefore might be underrepresented in lower-quality assemblies. We compared the repeat element content across all assemblies by annotating repeats using *RepeatMasker* [28], using the standard “avian” *Repbase* repeat element library [29]. All other settings for *RepeatMasker* were set to default values.

A previous study quantified Single Sequence Repeat (SSR; also known as microsatellite) content in both of these bird species based on analysis of the raw unassembled Illumina reads [26]. We repeated the analysis on the *de novo* and reference-guided assemblies for both species to assess if SSR content varied among genome assemblies compared to the raw reads (which might indicate the under-representation of SSRs in certain assemblies). We used *Palfinder* v0.02.03 [26] to identify SSRs across genome assemblies, with an SSR being classified as a stretch of 2–6mer tandem repeats that met a certain tandem repeat threshold: 6 tandem repeats for 2mers, 4 tandem repeats for 3mers, and 3 tandem repeats for 4mers, 5mers, and 6mers. For comparative purposes, we used the same methods to estimate SSR content in both reference genomes used, as well as the Turkey (*Meleagris gallopavo*; [30]) and the *Anolis* lizard (*Anolis carolinensis*; [31]) genomes.

### Analysis of GC isochore structure

To examine whether such relatively low coverage genome assemblies could provide information about genomic GC isochores, we compared patterns of regional variation in nucleotide composition (e.g “isochores”) between our reference-guided genomes and other high-quality vertebrate genomes. To do this, we estimated the standard deviation of GC content for genomic windows of varying sizes: 3-, 5-, 10-, 20-, 80-, 160-, and 320-kb. The expectation is that standard deviation will decrease as window sizes increase; based on a completely homogeneous genome, variation will halve as window sizes quadruples [32]. Deviations from this expectation indicate a genome with structural variation in GC content, as observed in mammals and birds but not in the *Anolis* lizard genome [33]. In addition, we randomly sampled 3- and 5-kb windows from the Chicken genome to match the sample size in the Clark's Nutcracker to determine whether the sample size of the dataset was representational of genome-wide estimate of GC structure at these spatial scales. Patterns in GC variation, and how it declines as window size changes, can quantify the heterogeneity of GC content in a genome. For example, a genome that has a large GC content standard deviation for larger windows has significant nucleotide composition heterogeneity at a large spatial scale, indicative of strong isochore structure. Multiple mammal, bird, and reptile genomes were used to compare the compositional structure of genomes among vertebrates.

### Variant analysis

We analyzed the relative frequencies of various types of heterozygous variants in the two bird genomes by mapping our quality-filtered Illumina reads back to the 1x reference-guided assemblies and by applying a Bayesian approach to determine the probability of heterozygosity at each position implemented in the Probabilistic Variant Detection tool in CLC Genomics Workbench. Heterozygous variants were filtered based on the following criteria: a minimum coverage of 4 reads, with at least two reads supporting a variant, and a variant probability of at least 80%. The analysis ignored non-specific matches, broken paired-end reads, and variants in non-specific regions, and required the presence of a variant in both the forward- and reverse-facing reads, and to expect a maximum of 2 variants per position. We further filtered these data to provide a more robust estimate of the heterozygosity using the following parameters and thresholds: read coverage greater than 5 reads, allele frequencies between 30% and 70%, forward and reverse reads both support the variant in at least 30% of the reads, and an average PHRED quality score of greater than 40.

### Mitochondrial genome assembly

Mitochondrial genome reads were extracted from all reads prior to genome assembly, and used to reconstruct the mitochondrial genomes of both species for use in divergence time estimation between our target species and species used as genome references for each of our targets. The mitochondrial genome of each bird was identified by using *blast* [34] to search for *de novo* assembled contigs using the consensus complete mitochondrial genome sequence from all members of the order Galliformes (Sage-Grouse), and a consensus for the family Corvidae (Clark's Nutcracker; Tables S1–S2). Contigs from the assembly that were matched by *blast* to the mitochondrial genome consensus sequences (of other previously sampled birds) were used to further assemble the mitochondrial genome. We created the assemblies by mapping the *blast* hits to the consensus mitochondrial genome sequence in CLC Genomics Workbench, using a mismatch cost  = 2, insertion cost  = 3, deletion cost  = 3, length fraction  = 0.5, and similarity fraction  = 0.8. The consensus sequence was then exported using a minimum coverage threshold of 1x. At positions where the threshold of low coverage was not met, an N ambiguity code was inserted. We note that a separate study has recently conducted similar analyses using these data and deposited on NCBI nearly identical results [35], and we therefore have not deposited our versions of these mitochondrial genome sequences in NCBI to avoid redundancy. We have, however, used our versions of these mitochondrial genomes for analysis because they were slightly more complete for some genes for the Sage-Grouse. Additionally, identification and removal of mitochondrial reads from the remaining data enable characterization of patterns solely from the nuclear genome of both species.

### Mitochondrial gene phylogeny and divergence estimates

To accurately date divergence times between our target species and those that we used as guides for assembly, we obtained additional mitochondrial genomes from NCBI. We chose taxa to represent most avian lineages, with diverse representatives of the Galliformes, Passeriformes, and several outgroups (n = 20 taxa; see Fig. 9 and Table S3), and specifically included taxa for which divergence times had been estimated previously [22]–[25]. Our phylogenetic analysis included sequences from 12 mitochondrial protein-coding genes (excluding ND6 and all non-coding loci; see Table S3 for NCBI accession numbers). Annotated sequences from the mitochondrial genome of the Chicken were used as a reference to align and trim sequences. Complete mitochondrial protein sequences were then aligned using *Geneious* 6.1.6 (Biomatters Ltd.), followed by minor manual adjustment, and were concatenated using *Sequence Matrix 1.7.8* [36]. Best-fit models of nucleotide evolution for each gene and codon position were estimated using Bayesian Information Criterion (BIC) in the program *PartitionFinder v1.1.1* [37]. The final alignment included a total of 10,845 bases for each species. A list of the best-fit models of nucleotide evolution used is included in the supporting materials (Table S4).

We estimated phylogenetic relationships using Bayesian Markov Chain Monte Carlo inference (BI) with all concatenated genes in *MrBayes version 3.2.1* [38]. Analyses were conducted using 10^7^ generations for each of two simultaneous runs, each with four chains (three heated and one cold) that were sampled every 1,000 generations. We estimated divergence times among taxa using BEAST 2 [39], [40], and used the consensus tree resulting from *MrBayes* as a starting guide tree for BEAST 2 analyses. Divergence estimation in BEAST 2 used the concatenated mitochondrial gene set, with an HKY substitution model, a lognormal relaxed clock model, and a Yule process tree prior. We constrained nodes using dates obtained from previous mitochondrial divergence time estimates [22]–[25]. A list of calibration points used in the analysis is given in the supporting materials (Table S5). Two independent analyses were run for 5 x 10^6^ generations, sampling every 1,000 generations. We used the program *Tracer* [39] to confirm if the analyses had reach convergence based on likelihood and parameter value stationarity, and based on this discarded the first 10% of generations from each run as burn-in. We used the program *TreeAnnotator v. 1.7.4* [39] to summarize parameter values of the samples from the posterior on the consensus tree.

## Results

### Genome *de novo* assemblies

**Figure 1:**
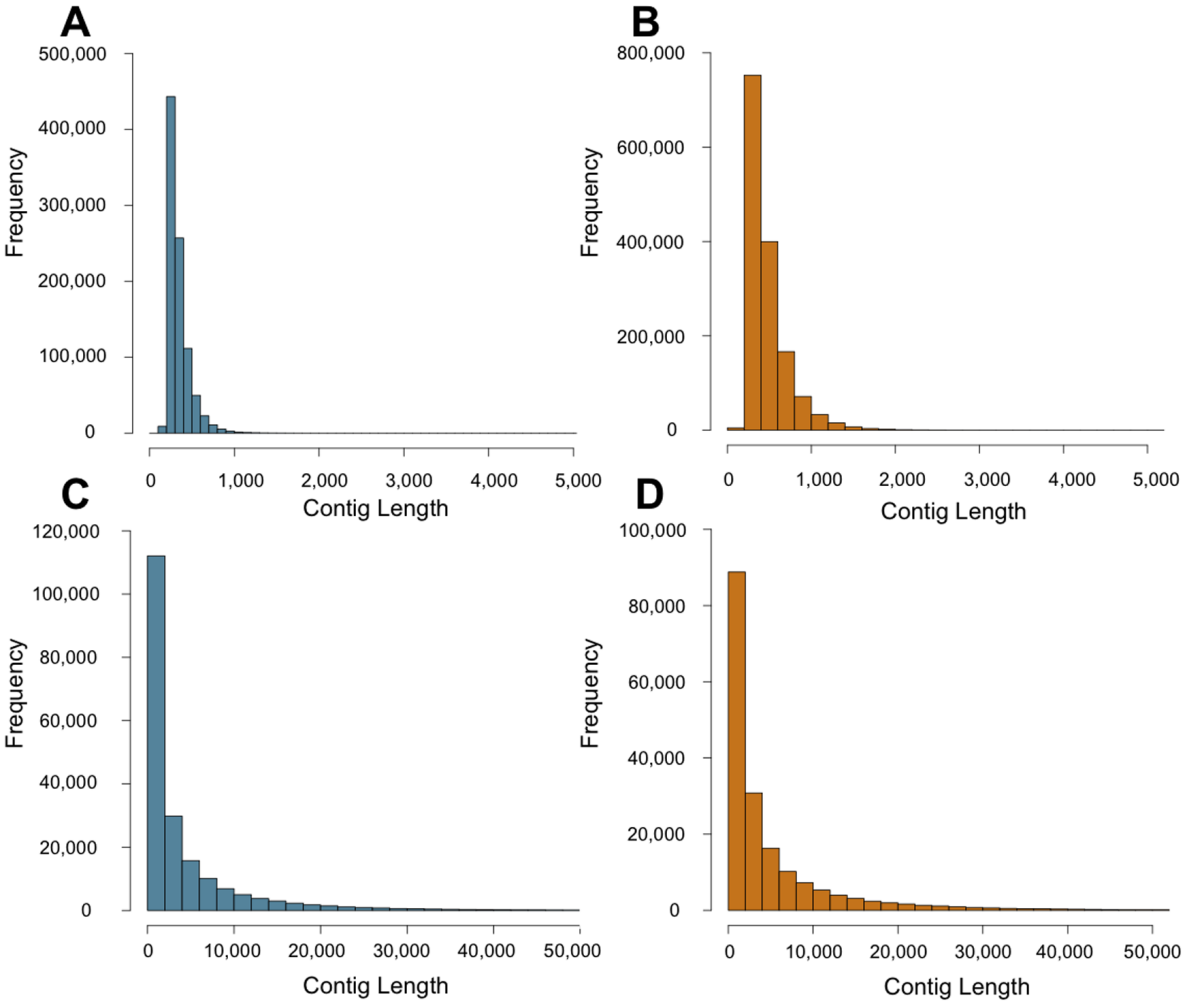
Genomic contig sizes based on various assembly strategies. Frequency histograms of contig sizes for (**A**) the Sage-Grouse *de novo* assembly, (**B**) the Clark's Nutcracker *de novo* assembly, (**C**) the Sage-Grouse reference-guided assembly (1x read coverage) split at (N)_500_ motifs, and (**D**) the Clark's Nutcracker reference-guided assembly (1x read coverage) split at (N)_500_.

**Figure 2:**
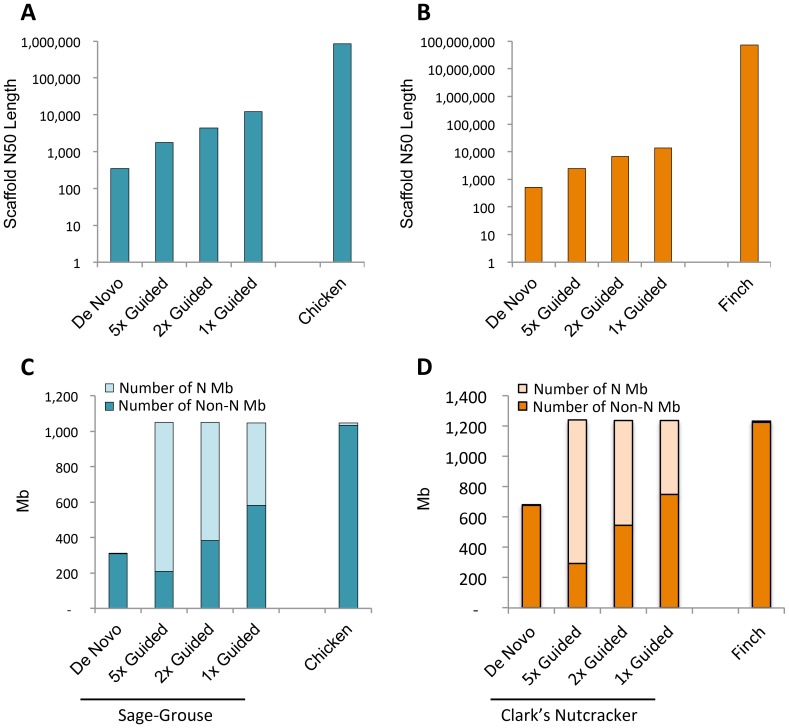
Comparison of N50 scaffold length and total assembly length for various assemblies. Histograms of the N50 scaffold length for new bird genomes with (N)_500_ motifs removed and total genome sizes for guide genomes. (**A**) N50 contig length for the Chicken reference genome, the *de novo* Sage-Grouse genome, and each of the guided assembly genomes. (**B**) N50 scaffold length for the Zebra Finch reference genome, the *de novo* Clark's Nutcracker genome, and each of the Clark's Nutcracker guided assembly genomes. Note that the y-axis scales differ between panels A and B. (**C**) Total genome sizes for the Chicken reference genome, *de novo* Sage-Grouse, and three guided Sage-Grouse genomes at different read coverage levels. (**D**) Total genome sizes for the Zebra Finch reference, *de novo* Clark's Nutcracker, and three guided Clark's Nutcracker genomes at different read coverage levels.

Assuming that the genome sizes of each species equaled the mean known genome size for their respective families (both 1.32 Gb [41]), our genome sampling represents approximately 3.53x genome coverage of the Sage-Grouse and 5.41x for Clark's-Nutcracker (Table 1). A summary of the numbers of reads, total bases, and estimated genome sizes are given in Table 1. The *de novo* assembly of the Sage-Grouse totaled 309,822,517 bp, comprising 914,239 scaffolded contigs (Fig. 1A; Table 2). Most contigs were less than 1,000 bp in length (Fig. 1A), and the N50 contig size was 343 bp (Fig. 2A). The assembly consisted of 31.6% Adenine (A), 18.5% Cytosine (C), 19.0% Guanine (G), and 30.9% Thymine (T). The *de novo* assembly of the Clark's Nutcracker totaled 679,286,238 bp, comprising 1,457,264 scaffolded contigs (Fig. 1B; Table 2). While most contigs were again less than 1,000 bp in length, contig sizes tended to be slightly larger in the Clark's Nutcracker than in the Sage-Grouse (Figs. 1A–B). This slight shift upward in contig size is also observed in the larger N50 contig size in the Clark's Nutcracker (503 bp; Fig. 2B), as well as a higher maximum contig size (18,041 bp). The assembly consisted of 29.5% (A), 20.5% (C), 20.8% (G), and 29.0% (T).

**Table 1:** Summary of raw genome sequence data used.

Species	Reads	Total Bp	Estimated genome size (Gb)	Estimated fold coverage
Sage-Grouse	39,582,844	4,662,514,211	1.32	3.53
Clark's Nutcracker	60,573,448	7,135,441,227	1.32	5.41

**Table 2:** Summary of genome assembly statistics from various assembly approaches.

	Sage-Grouse				Clark's Nutcracker			
		Reference-guided				Reference-guided		
	*De novo*	Coverage >1x	Coverage >2x	Coverage >5x	*De novo*	Coverage >1x	Coverage >2x	Coverage >5x
% N Bases	0.04	44.35	63.36	79.99	0.09	39.55	55.86	76.34
N50 - No Break	343	90,198,103	90,394,695	90,527,046	503	65,905,513	73,959,172	74,132,310
N50 - Break 500	–	12,125	4,447	1,804	–	13,369	6,765	2,409
Complete CEGs	0	12	0	0	4	76	20	1

The terms ‘no break’ and ‘break 500’ refer to whether or not contigs were broken up by deleting regions that contained stretches of 500 or more ambiguous (“N”) nucleotides, and CEGs refer to core eukaryotic genes.

### Reference-guided assemblies

The total length of reference-guided assemblies for the Sage-Grouse were over 1 Gb, approximating the length of the Chicken reference genome, though a large fraction of this sequence consisted of “N” ambiguities due to low coverage and/or the number of reads mapping to the reference falling below set thresholds (Fig. 2C). When genome segments containing stretches of at least 500 N bases were removed, most remaining contigs were longer than 1,000 bp, with many being 10,000 bp or greater in the 1x reference-guided genome (Fig. 1C); this trend is also clear from the larger N50 contig sizes observed in the reference-guided assemblies (Fig. 2A; Table 2). The reference-guided assemblies for the Clark's Nutcracker showed trends similar to the Sage-Grouse in having substantial numbers of ambiguous bases comprising the reference-guided assemblies (Fig. 2D). The contigs that resulted from splitting stretches of at least 500 N bp were predominantly greater than 1,000 bp in length, with some contigs longer than 30 kb in the 1x reference-guided genome (Fig. 1D); N50 contig sizes for all three reference-guided assemblies were greater than 1,000 bp (Fig. 2B; Table 2). The *de novo* assembly, all reference-guided assemblies, and a chromosome annotated version of the 1x reference-guided assembly are available for each species from the Dryad Digital Repository [42].

### Presence of CEGMA genes in assemblies

**Figure 3:**
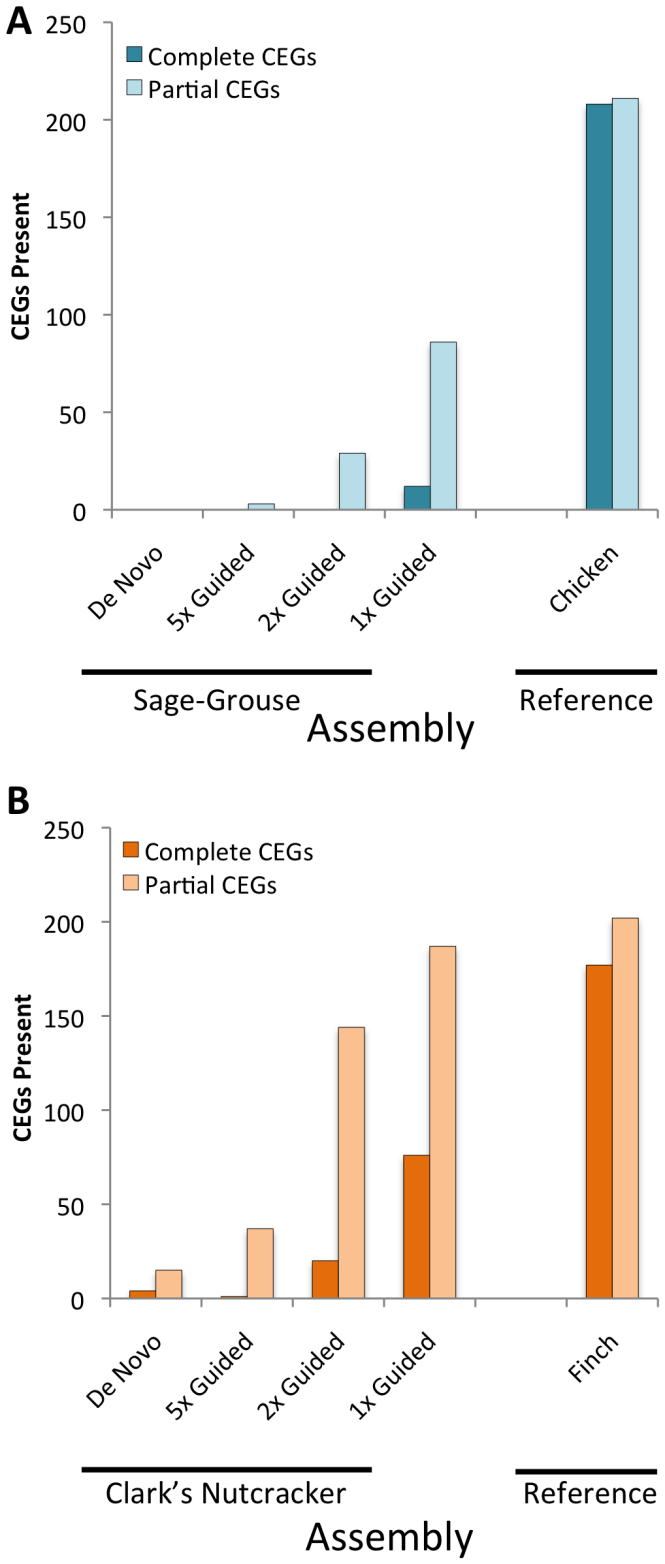
Comparison of Core Eukaryotic Genes identified in various new and reference genome assemblies. Histogram of the number of complete and partial ultraconserved CEGs obtained from the CEGMA pipeline. Maximum number of CEGs is 248. (**A**) The *de novo* assembly and three guided genome assemblies for the Sage-Grouse at different read depth thresholds, plus the guide genome the Chicken. (**B**) The *de novo* assembly and three guided genome assemblies for the Clark's Nutcracker at different read depth thresholds, plus the guide genome the Zebra Finch.

We used CEGMA to assess the completeness of assemblies with respect to protein coding regions in both the *de novo* and the reference-guided genomes. *De novo* assemblies for both species had consistently far lower numbers of CEGMA genes identified (either partial or complete) compared to the reference-guided assemblies (Figs. 3A–3B), with the 1x reference-guided assemblies containing the most CEGMA genes (Fig. 3). It is notable that we observed substantial increases in CEGMA gene content with relatively minor changes in assembly length among the reference-guided assemblies with different read depth cutoffs (Figs. 2A–2B and 3). Comparing the two species, the Clark's Nutcracker assemblies showed systematically higher recoveries of CEGMA genes than the Sage-Grouse (Fig. 3), which parallels the higher coverage, longer contigs, and larger non-ambiguous assemblies in the Clark's Nutcracker.

### Repeat element content

**Figure 4:**
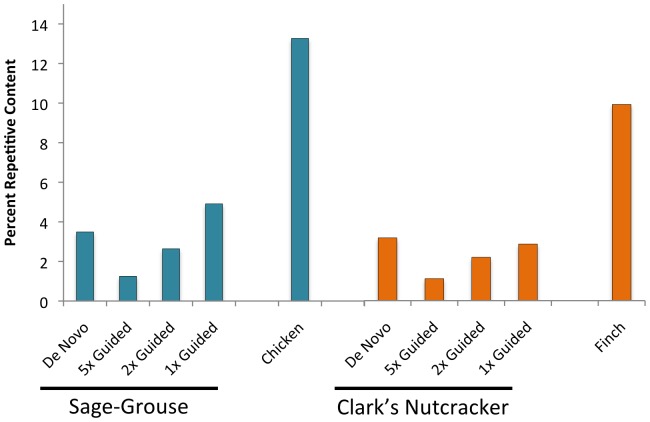
Percent of the genome identified as repetitive elements by RepeatMasker. Histograms of percent repetitive content for all assemblies and the reference genomes of both species. Repetitive content was estimated using RepeatMasker.

Because repetitive elements are notoriously difficult to assemble, we compared the abundance of repetitive elements in various genome assemblies. *A priori*, we assumed that poorly assembled or less completely assembled genomes would contain fewer annotated repetitive elements than higher-quality and more complete genomes. In general, this expectation holds in comparisons between the reference genomes and our *de novo* and reference-guided assembly genomes (Fig. 4). In the Sage-Grouse, the genome assembly with the most repetitive content was the 1x reference-guided assembly, followed by the *de novo* assembly (Fig. 4). In the Clark's Nutcracker, which also had substantially more raw read data, the *de novo* assembly contained the greatest repeat element fraction compared to the reference-guided assemblies (Fig. 4). Neither the *de novo* or reference-guided assemblies, however, contained a similar amount of repeat elements as that in the respective reference genomes, indicating that much of the unassembled parts of the Clark's Nutcracker and the Sage-Grouse genomes may represent a biased failure to incorporate repeat elements.

### Simple sequence repeat content

**Figure 5:**
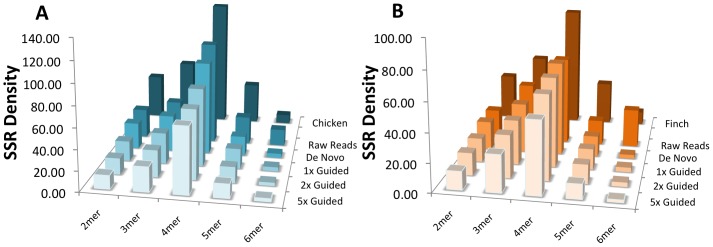
Genomic simple sequence repeat (SSR) density in raw reads and various genome assemblies. Histograms of the simple sequence repeat (SSR) density of sequence is given for raw sequence reads, each of the assembly genomes, and reference genomes for (**A**) the Sage-Grouse and (**B**) the Clark's Nutcracker. Density for each motif length is the number of motif loci per Mb.

**Figure 6:**
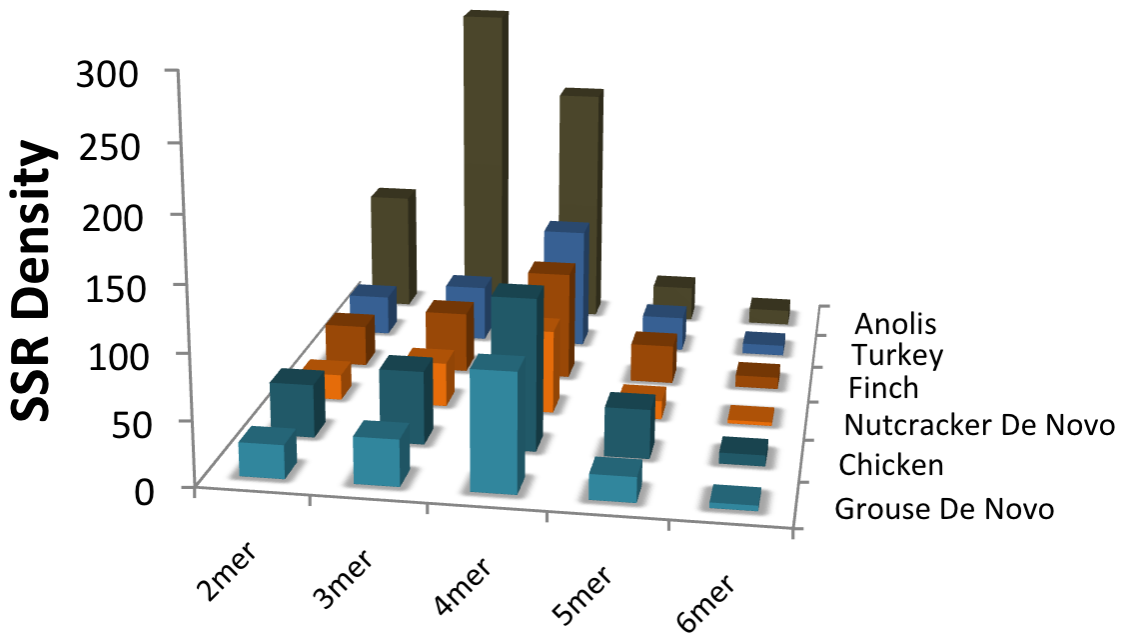
Genomic simple sequence repeat (SSR) density across select amniote vertebreate genomes. Histograms of SSR density for each *de novo* assembly and its respective reference genome, and for the Turkey (*Meleagris gallopavo*) and the Anolis Lizard (*Anolis carolinensis*) genome assemblies. Density for each motif length is the number of motif loci per Mb.

We estimated simple sequence repeat (SSR, or microsatellite) content of various assemblies to further examine qualitative and quantitative ways in which the *de novo* and reference-guided assemblies differed, and how they compared to high quality reference genomes. Because raw reads can also be used to identify SSR content [26], we included analysis of unassembled reads in comparisons. Analogous to our findings with general repeat elements, we determined that the *de novo* assemblies contain the highest abundances of SSRs (Fig. 5). Also, unlike the general repeat element analysis, the SSR content estimates from the *de novo* assemblies are relatively similar to estimates in the high quality reference genomes, although the estimates derived from raw reads proved to be even better approximations to SSR densities observed in high-quality reference genomes (Fig. 5). Comparative analysis of SSR content across bird species indicates that genomic SSR content is relatively conserved among avian genomes, except for some variance in the abundance of 2–4mers (Fig. 6). In contrast to the conservation of the SSR landscape across bird species, the SSR landscape changes extensively between birds and the *Anolis* lizard, particularly in the abundance of 2–4mer SSRs (Fig. 6).

### Genomic GC-isochore structure

**Figure 7:**
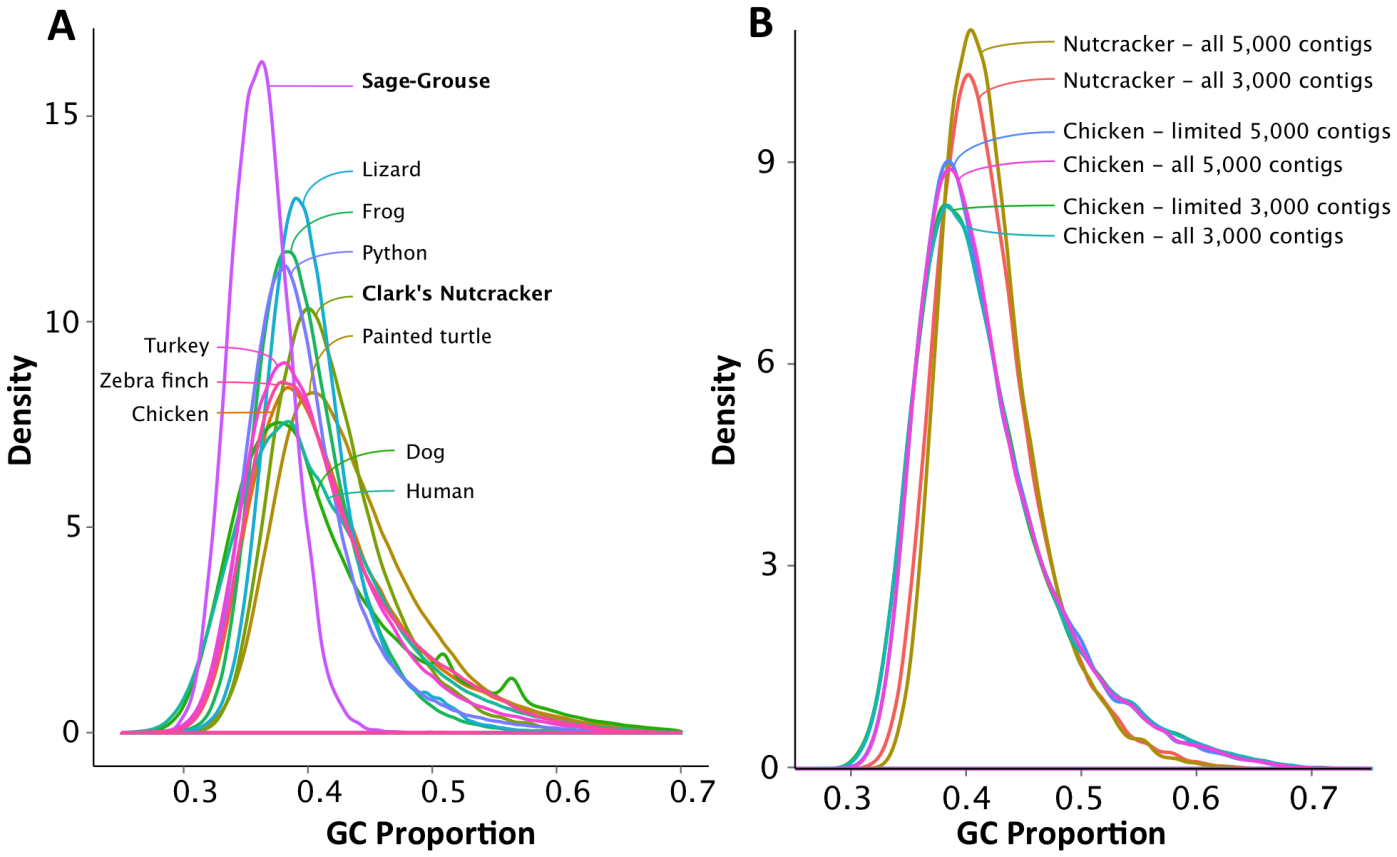
Genomic GC isochore structure among amniote vertebrates, and in draft genomes. (**A**) GC isochore structure plot of 1x guided assemblies for both bird species, their reference genomes, and other select amniote vertebrate genomes using a 3 kb window size. (**B**) GC isochore structure plot comparison of 1x the Clark's Nutcracker guided assembly and the reference the Chicken genome. All contigs at both a 3,000 and a 5,000 bp window were used for the Clark's Nutcracker (n = 73,158 and n = 30,090 contigs respectively). All contigs (referred to as “all” in figure) or a random selection equal to the number of contigs in the Clark's Nutcracker assembly (“limited”) for both the 3,000 and 5,000 bp window were used in the comparison.

Comparison of genomic GC-isochore structure across vertebrates is typically thought to require very well-assembled genomes, because it requires long contiguous regions of genome assemblies. We were interested to test if reference-guided genomes could be used for estimation of GC-isochore structure, and if they produced results that were reasonable compared to other related bird species. Overall, the *de novo* genome assemblies for both bird species did not contain enough contigs to adequately estimate GC content variation at large spatial scales. The 1x reference-guided assembly yielded the highest number of contigs at each window size and was used for subsequent comparison with other vertebrate genomes and with a randomly-sampled, proportionally reduced representation 3- and 5-kb contig sample from the Chicken. The distribution of GC content for the Sage-Grouse differed considerably from any other vertebrate genome, most likely because the estimate of GC isochore structure was unreliable for this species' assembly, which also had very low genome coverage and small contig sizes. However, the distribution for the Clark's Nutcracker was much more similar to that of other vertebrates, yet differed from the other bird genomes in having a slightly higher GC content and a more narrow distribution (Fig. 7A). To examine whether these differences are the consequence of the smaller sample sizes (73,158 and 35,090 3- and 5-kb windows, respectively, versus 338,120 and 202,814 3- and 5-kb windows, respectively, in the Chicken), we used a random subset of the Chicken genome windows to match the sample sizes of genomic windows available for the Clark's Nutcracker. We compared the GC distributions between the full and reduced sample sizes in the Chicken and found no difference (Kolmogorov-Smirnov test: *p* = 0.5026 for the 3-kb window size comparison and *p* = 0.8398 for the 5-kb window size comparison), indicating that such a reduced data set of genomic windows provides an adequate representation of the genome-wide GC content distribution at 3- and 5-kb window sizes. This, together with the inference of no clear assembly bias in GC content (Table S6), indicate that the GC distribution of the Clark's Nutcracker at the 3- and 5-kb window sizes is expected to accurately reflect the genomic GC content variation at these various spatial scales (Fig. 7B).

### Variant detection

**Figure 8:**
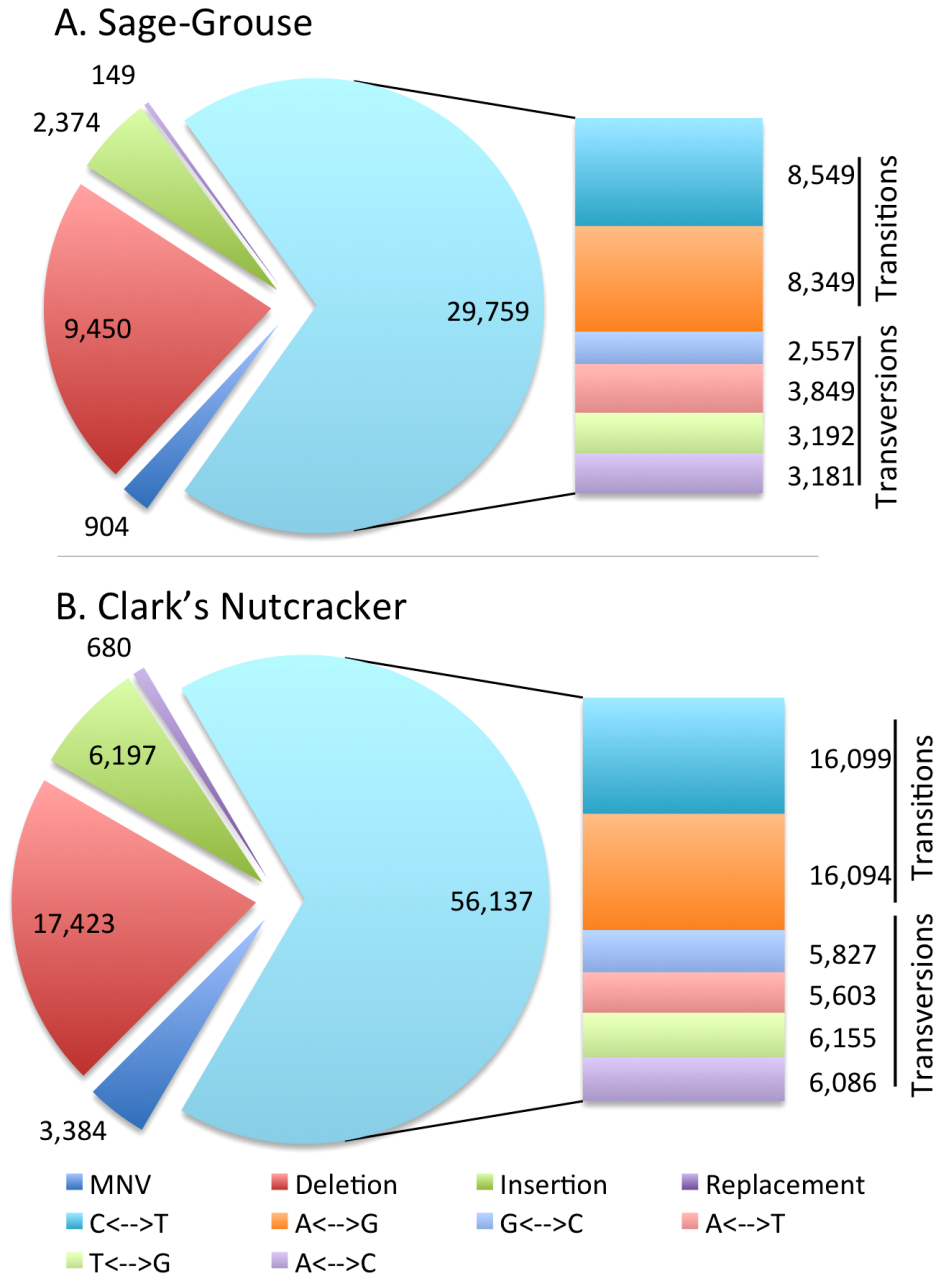
Heterozygous variant composition for the Sage-Grouse and the Clark's Nutcracker. Pie chart includes Single Nucleotide Variants (SNV), Multiple Nucleotide Variants (MNV), Insertions, Deletions, and Replacements. SNVs are further annotated in a bar graph form according to all possible transitions. Key provides color-coding for each variant.

We examined variants with reasonable coverage thresholds to compare the relative frequencies of observed types of heterozygous variants between species. Overall, the relative levels of heterozygous variants for each bird were approximately equal, despite the Clark's Nutcracker having nearly double the number of each variant type when compared to the Sage-Grouse; this was expected due to the lower number of sites that met the criteria for calling heterozygous variants in the Sage-Grouse. Single nucleotide variants (SNVs) were most frequently observed with deletions also occurring regularly, and SNVs that represented transitions were much more frequently observed than transversions (Fig. 8). Multiple nucleotide variants (MNVs), insertions, and replacements were represented in lower frequencies in both genomes, but were similar in relative frequencies among the two species (Fig. 8).

**Figure 9:**
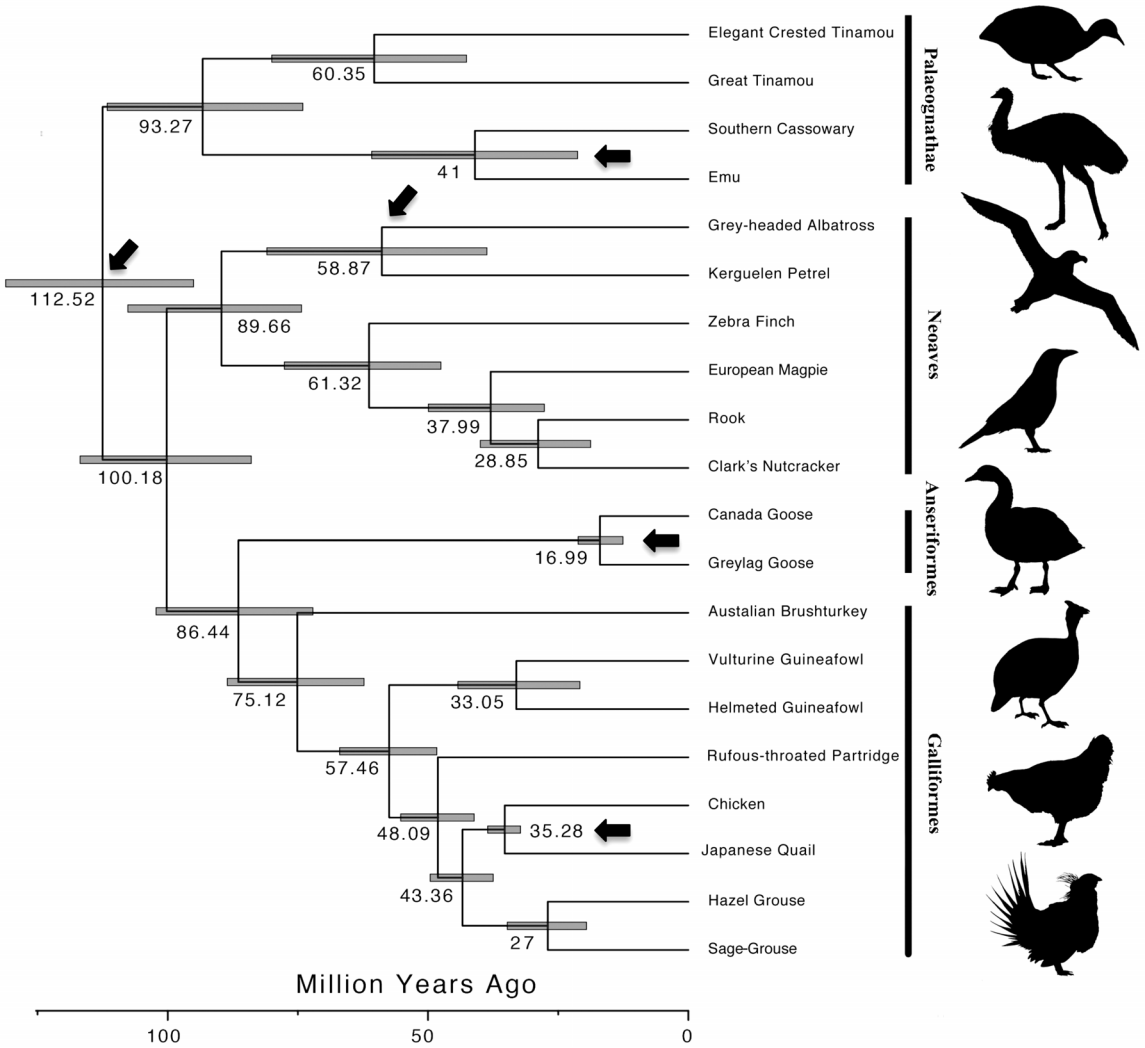
Estimated divergence times among birds, including focal and reference genome species. Bayesian relaxed clock estimate of divergence times among several bird lineages based on 12 mitochondrial protein-coding genes, with 95% credibility intervals shown as shaded bars at nodes. Dark arrows represent calibration points used in the analysis.

### Mitochondrial genome assemblies

The reference-guided mitochondrial genome assembly for the Sage-Grouse was incomplete and was likely related to the lower coverage available for this species; 59.08% of the mitochondrial genome was unresolved (and represented as ambiguities), and three of the 12 mitochondrial protein-coding loci used for phylogenetic analysis were essentially absent (and the remaining nine contained some ambiguous regions). Despite this partial assembly, these data provided an ample number of aligned sites to conduct phylogenetic analyses. The reference-guided mitochondrial genome for the Clark's Nutcracker was much more complete than the Sage-Grouse. Across the entire mitochondrial genome, only 8.69% of sites were ambiguous (“N”s). For the Clark's Nutcracker, all 12 protein-coding mitochondrial genes used for phylogenetic analysis were present and contained no ambiguous bases. Annotated versions of the assemblies are available from the Dryad Digital Repository [42]. Mitochondrial genome assembly and annotation was therefore more complete for the Clark's Nutcracker than for the Sage-Grouse, which may due to the relative amount of data combined with the density of mitochondria in the different tissue sources used for DNA extraction: blood in the case of the Sage-Grouse versus muscle tissue in the case of the Clark's Nutcracker [35].

### Mitochondrial phylogeny and divergence dating of birds

Using the newly assembled mitochondrial genomes, we were able to estimate the phylogenetic relationships of the Clark's Nutcracker and the Sage-Grouse, as well as divergence times between these species and several other species of birds, including the two species used as reference genomes for guided assemblies. The Bayesian analysis recovered four major clades among the species sampled, which correspond to the major groups of birds, and all nodes received strong support (>95% posterior). We inferred that the Clark's Nutcracker formed a clade with the Rook (*Corvus frugilegus*), while the Sage-Grouse was nested in the Galliformes as sister species to the Hazel Grouse (*Bonasa bonasia*), and our divergence time estimates resulted in divergence ages similar to those of previous studies (Fig. 9; [22]–[25]). Most importantly, we estimated that the Sage-Grouse split from its common ancestor with the Hazel Grouse approximately 27 million years ago (mya), while it split from the Chicken (*Gallus*) about 43 mya, and that the Clark's Nutcracker diverged from its common ancestor with the Rook approximately 28 mya and from the Zebra Finch approximately 61 mya.

## Discussion

Our results demonstrate that substantial information can be extracted from lower-coverage genomic sampling projects, and that reference-guided assemblies provide much better representation of biologically important regions than *de novo* assemblies when genome coverage is low. We were surprised that reference-guided assembly approach was quite successful despite substantial divergence between target species and reference genome species (∼40–60 mya; Fig. 9), and with fairly low levels of sequencing coverage (Table 1). While we suggest that higher coverage is preferable, our results provide an exciting proof of concept for an economical strategy to increase the diversity of vertebrate genome resources by using reference-guided assembly approaches. This strategy would be particularly useful for species that are somewhat closely related to those for which high-quality reference genomes are available. Such reference-guided low-coverage genomes do indeed fall short of the completeness of information contained in high-quality *de novo* assembled genomes, although our results indicate that compared to an alternative of having no information at all for a species, or to a highly fragmented *de novo* assembly from low-coverage data, reference-guided assemblies are capable of providing substantial biological information about the genome of a species at low cost.

While reference-guided genomes do appear to contain large amounts of biological information, the accuracy of this information is unknown, and probably dependent on the type of feature and the divergence between target and reference species. For example, estimates of most protein-coding genes are likely accurate given their conserved nature. More rapidly diverging genomic features or regions, such as transposable elements or other non-coding regions, may be more prone to inaccuracies in reference-guided assemblies. These inaccuracies will also increase with divergence between reference-target species, which may indeed lead to spurious contigs or nucleotide stretches that are not present in the actual garget genome. Thus, reference-guided genome estimates should be applied with the understanding that they may indeed be prone to inaccuracies and error, depending on reference-target sequence divergence. For this reason, it is also not wise to use one reference-guided assembly as a reference for a second reference-guided assembly, because errors and inaccuracies in assembly from one would be both perpetuated and compounded.

In both bird species analyzed here, reference-guided assemblies provided more complete representation of some important genomic features compared to *de novo* assemblies. The greatest difference in content among alternative assemblies was the number of CEGMA genes identified, with our *de novo* assemblies finding extremely few and reference-guided assemblies finding orders of magnitude more as coverage thresholds were lowered. This indicates that reference-guided approaches may be particularly useful for establishing genomic resources for gene-centric analyses. Repetitive elements tend to pose a particular challenge to *de novo* genome assembly in vertebrates [43], and we expected repetitive element content to be higher (and more similar to reference genomes) in reference-guided versus *de novo* assemblies. This was not necessarily the case in our results, however, and, instead, both approaches seem to under-represent genomic repetitive element content, indicating that that these repetitive elements may be just as challenging for mapping (in reference-guided assembly) as they are for *de novo* assembly. Having more closely related reference genomes may substantially improve how well repeat element regions are assembled, as the ability to use a reference-guided approach to assemble these regions may be highly dependent on the degree of recent activity of repeat elements in a particular lineage. In contrast to major differences in repeat element content between new and reference genomes, and among assembly approaches, SSR estimates show little variation across these comparisons of different genome assembly approaches for a particular species (Fig 5). This finding also confirms the utility of analyses that have quantified SSR density and diversity using raw reads [26], and indicates that read assembly gives no major advantage for identification and estimation of abundance of SSR loci on a genome-wide scale.

It is well established that avian genomes contain substantially less identifiable repetitive content than other vertebrate genomes, and are relatively depauperate in simple sequence repeats (SSRs) and transposable elements [1], [44]. Comparisons of the SSR content of avian and lizard genomes support this, confirming that bird genomes contain substantially less SSR content than does the lizard genome (Fig. 6); this trend was also observed in analogous comparisons to a snake genome sample [26]. It has been hypothesized that SSR evolution and turnover has been particularly slow in non-mammalian vertebrates [2], which is consistent with our findings of highly similar abundances of SSR loci across all bird genomes that we examined (Fig. 6), although this and other studies suggest this may not be the case in squamate reptiles like the *Anolis* lizard [45], [46].

Given previous evidence that the *Anolis* lizard essentially lacks the genomic GC-isochore structure present in birds and mammals [33], interest in understanding the evolutionary dynamics of GC-isochore structure across vertebrates has increased [33], [45], [47], [48]. Isochore structure is challenging to study with less than high-quality genome assemblies because it requires relatively long assembled regions of the genome. We therefore tested if reference-guided assemblies might provide a cost-effective alternative to the generation of high-quality genome assemblies for developing genomic resources for analysis of GC-isochore structure. While the sample sizes of windows were too small (20 windows of 320-kb in the Clark's Nutcracker) to confidently estimate variation in GC content at large spatial scales, we were able to estimate GC structure at smaller scales using the reference-guided assemblies. While this approach does not capture the full extent of isochore structure in a genome, we have observed previously that smaller windows still provide insight into GC content variation, especially when compared across vertebrates (Fig. 7A; [33]). We found that variation in GC content at 3 kb and 5 kb window sizes for the Clark's Nutcracker resembled the structure known for other bird genomes (Fig. 7A). More interestingly, based on our sampling experiment, the Clark's Nutcracker assembly may be complete enough to capture the GC heterogeneity at these smaller spatial scales (Fig. 7B). This finding suggests that low (and therefore less-expensive) genome sequencing coverage, combined with a reference guided assembly approach, may hold great promise for economically providing novel insight into genomic GC heterogeneity across a large diversity of vertebrates.

Using reference-guided assemblies, we were able to establish that the relative proportions of certain variant classifications were very similar in both bird species, although the Clark's Nutcracker typically had about twice the number of each variant type (Fig. 8). This corresponds to the approximate genome coverage being about twice as high for the Clark's Nutcracker (Table 1). Thus, low coverage genome assemblies do appear to be useful for analysis of possible shifts in the proportions of certain types of heterozygous variants, and potentially for understanding shifts in genomic mutation spectra among lineages.

Among amniote vertebrates, birds are notable for their high levels of karyotypic conservation [49]–[51], genomic synteny [52], [53], and low repeat element content [1], [54]. All these traits make bird genome assembly using *de novo* and reference-guided approaches more tractable, and indicate that among vertebrates, bird genomes may be a best-case scenario for the performance of reference-guided assembly approaches. It would therefore be interesting to investigate the utility of such lower-coverage reference-guided (versus *de novo*) assembly approaches in other lineages, such as mammals or non-avian reptiles. These lineages may have less conserved synteny and higher repeat element content, which implies that the amount of information available from a reference-guided approach may be more limited, and that the approach may only work well for more closely-related reference-target species pairs.

Until recently, only two high-quality and well-annotated bird genomes were available, the Chicken and the Zebra Finch [1], [3], yet additional bird genomes have begun to emerge [4], [30], [55]–[61]. Soon there will be approximately 50 additional high quality bird genomes completed as part of a Beijing Genomics – Genome 10K initiative (Erich Jarvis, pers. comm.). With so many diverse high-quality reference genomes available for birds expected in the near future, the reference-guided approach we test here may provide an attractive means of massively increasing knowledge of bird genome diversity with great economy. It is also notable that neither of the two bird species (or members of the same genera) will be included in these new 50 bird genomes, indicating that genome resources developed here will be highly useful and unique for the foreseeable future.

Not surprisingly, low-coverage reference-guided genome assemblies contain far less information than high-quality *de novo* assembled genomes. What is surprising is that such low-coverage reference-guided assemblies may yield substantial information about the genome of a species compared to a *de novo* assembly using the same data. Thus, approaches using low-coverage reference-guided assemblies, as well as other sample-sequencing approaches that sample <1x genome coverage [26], [46], [62], [63] hold strong potential to contribute novel insight into vertebrate genomic diversity decades before it is feasible to obtain high-quality genomes from a large number of vertebrates. Such approaches may also be useful for initial surveys of genomic diversity across the tree of life, thereby guiding larger-scale, high-quality genome sampling of particular species that show genomic characteristics and features that are biologically interesting based on such preliminary studies.

## Supporting Information

Table S1 **Species and NCBI accessions used to guide the Sage-Grouse mitochondrial genome reconstruction.**
(DOCX)

Table S2 **Species and NCBI accessions used to guide the Clark's Nutcracker mitochondrial genome reconstruction.**
(DOCX)

Table S3 **Species and NCBI accessions used for phylogeny and divergence estimation.**
(DOCX)

Table S4 **Best-fit models of nucleotide evolution for mitochondrial genes used in phylogenetic analyses.**
(DOCX)

Table S5 **Calibration points used in divergence time analysis.**
(DOCX)

Table S6 **Percent GC in new (and reference) genome assemblies.**
(DOCX)
