# Supplementary material for: Two Low Coverage Bird Genomes and a Comparison of Reference-Guided versus De Novo Genome Assemblies
Source: PLoS One. 2014 Sep 5;9(9):e106649. doi: 10.1371/journal.pone.0106649 (PMC4156343; doi:10.1371/journal.pone.0106649)
Supplement: Table S1 — Species and NCBI accessions used to guide the Sage-Grouse mitochondrial genome reconstruction. (DOCX) [file pone.0106649.s001.docx]

**Table S1. Species and NCBI accessions used to guide the Sage-Grouse mitochondrial genome reconstruction.**

| **Species** | **NCBI Accession Number** |
| --- | --- |
| *Acryllium vulturinum* | NC014180 |
| *Alectoris chukar* | FJ752426 |
| *Alectura lathami* | NC007227 |
| *Arborophila gingica* | FJ752425 |
| *Arborophila rufipectus* | FJ194942 |
| *Arborophila rufogularis* | NC020584 |
| *Bambusicola fytchii* | FJ752423 |
| *Bambusicola thoracica* | EU165706 |
| *Coturnix chinensis* | AB073301 |
| *Coturnix japonica* | AP003195 |
| *Crossoptilon auritum* | JF937589 |
| *Crossoptilon crossoptilon* | HQ891119 |
| *Francolinus pintadeanus* | EU165707 |
| *Gallus gallus* | NC001323 |
| *Gallus lafayettei* | AP003325 |
| *Gallus sonneratii* | AP006741 |
| *Gallus varius* | AP003324 |
| *Ithaginis cruentus* | JF921875 |
| *Lophophorus lhuysii* | GQ871234 |
| *Lophophorus sclateri* | FJ752432 |
| *Lophura ignita* | AB164627 |
| *Lophura nycthemera* | EU417810 |
| *Meleagris gallopavo* | EF153719 |
| *Numida meleagris* | NC006382 |
| *Pavo muticus* | EU417811 |
| *Perdix dauurica* | FJ752431 |
| *Phasianus colchicus* | FJ752430 |
| *Phasianus versicolor* | AB164626 |
| *Polyplectron bicalcaratum* | EU417812 |
| *Pucrasia macrolopha* | FJ752429 |
| *Syrmaticus ellioti* | AB164624 |
| *Syrmaticus humiae* | AB164625 |
| *Syrmaticus reevesii* | AB164623 |
| *Syrmaticus soemmerringi* | AB164622 |
| *Tetraophasis obscurus* | JF921876 |
| *Tetraophasis szechenyii* | FJ752428 |
| *Tetrastes bonasia* | NC020591 |
| *Tragopan temminckii* | FJ752427 |
