# Supplementary material for: Two Low Coverage Bird Genomes and a Comparison of Reference-Guided versus De Novo Genome Assemblies
Source: PLoS One. 2014 Sep 5;9(9):e106649. doi: 10.1371/journal.pone.0106649 (PMC4156343; doi:10.1371/journal.pone.0106649)
Supplement: Table S2 — Species and NCBI accessions used to guide the Clark's Nutcracker mitochondrial genome reconstruction. (DOCX) [file pone.0106649.s002.docx]

**Table S2. Species and NCBI accessions used to guide the Clark’s Nutcracker mitochondrial genome reconstruction.**

| **Species** | **NCBI Accession Number** |
| --- | --- |
| *Lanius tephronotus* | JX486029 |
| *Cyanopica cyanus* | JN108020 |
| *Corvus frugilegus* | NC002069 |
| *Urocissa erythrorhyncha* | JQ423932 |
| *Podoces hendersoni* | GU592504 |
| *Pica pica* | HQ915867 |
| *Oriolus chinensis* | JQ083495 |
