# Supplementary material for: Two Low Coverage Bird Genomes and a Comparison of Reference-Guided versus De Novo Genome Assemblies
Source: PLoS One. 2014 Sep 5;9(9):e106649. doi: 10.1371/journal.pone.0106649 (PMC4156343; doi:10.1371/journal.pone.0106649)
Supplement: Table S3 — Species and NCBI accessions used for phylogeny and divergence estimation. (DOCX) [file pone.0106649.s003.docx]

**Table S3. Species and NCBI accessions used for phylogeny and divergence estimation.**

| **Species** | **NCBI Accession Number** |
| --- | --- |
| *Corvus frugilegus* | NC002069 |
| *Dromaius novaehollandiae* | NC002784 |
| *Tinamus major* | NC002781 |
| *Eudromia elegans* | NC002772 |
| *Casuarius casuarius* | NC002778 |
| *Branta canadensis* | NC007011 |
| *Pterodroma brevirostris* | NC007174 |
| *Alectura lathami* | NC007227 |
| *Diomedea chrysostoma* | AP009193 |
| *Anser anser* | NC011196 |
| *Pica pica* | HQ915867 |
| *Coturnix japonica* | AP003195 |
| *Numida meleagris* | NC006382 |
| *Acryllium vulturinum* | NC014180 |
| *Arborophila rufogularis* | NC020584 |
| *Tetrastes bonasia* | NC020591 |
| *Gallus gallus* | NC001323 |
| *Taeniopygia guttata* | NC007897 |
