# Supplementary material for: Two Low Coverage Bird Genomes and a Comparison of Reference-Guided versus De Novo Genome Assemblies
Source: PLoS One. 2014 Sep 5;9(9):e106649. doi: 10.1371/journal.pone.0106649 (PMC4156343; doi:10.1371/journal.pone.0106649)
Supplement: Table S4 — Best-fit models of nucleotide evolution for mitochondrial genes used in phylogenetic analyses. (DOCX) [file pone.0106649.s004.docx]

**Table S4. Best-fit models of nucleotide evolution for mitochondrial genes used in phylogenetic analyses.**

| **Gene** | **Codon Position** | **Model** | **Gene** | **Codon Position** | **Model** |
| --- | --- | --- | --- | --- | --- |
| **ATP6** | 1st | GTR+Γ | **ND1** | 1st | JC |
| **ATP6** | 2nd | GTR+Γ | **ND1** | 2nd | JC |
| **ATP6** | 3rd | HKY+I+Γ | **ND1** | 3rd | HKY |
| **ATP8** | 1st | HKY+Γ | **ND2** | 1st | HKY+Γ |
| **ATP8** | 2nd | HKY+Γ | **ND2** | 2nd | HKY+Γ |
| **ATP8** | 3rd | HKY+Γ | **ND2** | 3rd | HKY+Γ |
| **CO1** | 1st | HKY+I+Γ | **ND3** | 1st | HKY+Γ |
| **CO1** | 2nd | HKY+I+Γ | **ND3** | 2nd | HKY+Γ |
| **CO1** | 3rd | HKY+Γ | **ND3** | 3rd | HKY+Γ |
| **CO2** | 1st | HKY+Γ | **ND4** | 1st | HKY+Γ |
| **CO2** | 2nd | HKY+Γ | **ND4** | 2nd | HKY+Γ |
| **CO2** | 3rd | GTR+I+Γ | **ND4** | 3rd | GTR+Γ |
| **CO3** | 1st | HKY+Γ | **ND4L** | 1st | HKY+Γ |
| **CO3** | 2nd | HKY+Γ | **ND4L** | 2nd | HKY+Γ |
| **CO3** | 3rd | HKY+Γ | **ND4L** | 3rd | HKY+I+Γ |
| **CytB** | 1st | HKY+I+Γ | **ND5** | 1st | HKY+Γ |
| **CytB** | 2nd | HKY+I+Γ | **ND5** | 2nd | HKY+Γ |
| **CytB** | 3rd | HKY+Γ | **ND5** | 3rd | HKY+I+Γ |
