# Supplementary material for: Two Low Coverage Bird Genomes and a Comparison of Reference-Guided versus De Novo Genome Assemblies
Source: PLoS One. 2014 Sep 5;9(9):e106649. doi: 10.1371/journal.pone.0106649 (PMC4156343; doi:10.1371/journal.pone.0106649)
Supplement: Table S5 — Calibration points used in divergence time analysis. (DOCX) [file pone.0106649.s005.docx]

**Table S5. Calibration points used in the divergence time analysis.**

|  | **Distribution** | **Mean (mya)** | **StDev (mya)** |
| --- | --- | --- | --- |
| ***Anser-Branta*** | Normal | 14.5 | 2.7 |
| **Archosauria** | Normal | 243 | 3.6 |
| **Aves** | Normal | 93.5 | 17 |
| ***Coturnix-Gallus*** | Normal | 35 | 1.7 |
| **Neoaves** | Normal | 91.9 | 7.8 |
