# Supplementary material for: Two Low Coverage Bird Genomes and a Comparison of Reference-Guided versus De Novo Genome Assemblies
Source: PLoS One. 2014 Sep 5;9(9):e106649. doi: 10.1371/journal.pone.0106649 (PMC4156343; doi:10.1371/journal.pone.0106649)
Supplement: Table S6 — Percent GC in new (and reference) genome assemblies. (DOCX) [file pone.0106649.s006.docx]

**Table S6. Percent GC in new (and reference) genome assemblies.**

|  | **Assembly** | **Percent GC Content** |
| --- | --- | --- |
| **Sage-Grouse** | *De novo* | 37.5 |
|  | 1x Guided | 38.9 |
|  | 2x Guided | 38.6 |
|  | 5x Guided | 38.0 |
|  | Chicken | 41.8 |
| **Clark’s Nutcracker** | *De novo* | 41.4 |
|  | 1x Guided | 41.4 |
|  | 2x Guided | 41.6 |
|  | 5x Guided | 42.0 |
|  | Zebra Finch | 41.4 |
